# Long-term exposure to ambient PM_2.5_ and mortality: A comparison of between-subjects and within-subjects survival analysis

**DOI:** 10.1097/EE9.0000000000000378

**Published:** 2025-03-14

**Authors:** Martin Resua Rojas, Julien Vachon, Elhadji Anassour Laouan Sidi, Claudia Blais, Ying Liu, Audrey Smargiassi, Stephane Buteau

**Affiliations:** a Institut national de santé publique du Québec, Montreal, Quebec, Canada; b Department of Environmental and Occupational Health, School of Public Health, University of Montreal, Montreal, Quebec, Canada; c Center for Public Health Research (CReSP), University of Montreal and CIUSSS du Centre-Sud-de-l’Ile-de-Montreal, Montreal, Quebec, Canada; d Faculty of Pharmacy, Laval University, Quebec, Quebec, Canada

**Keywords:** Air pollution, Mortality, Cohort, Fine particles, Self-controlled

## Abstract

**Background:**

Cohort studies have reported positive associations between long-term exposure to ambient fine particles (PM_2.5_) and mortality. However, there is heterogeneity across results that may be due to unmeasured and residual confounding. We aim to compare two distinct types of analysis to examine whether long-term exposure to air pollution is associated with all-cause and ischemic heart disease (IHD) mortality: (i) a traditional survival analysis that contrasts different individuals at similar times, and (ii) a self-controlled design that controls for time-invariant confounders by contrasting the same person at different times.

**Methods:**

We used an open population-based cohort created from health administrative databases. The cohort included all adults older than 20 years living in the province of Quebec, Canada, between 2002 and 2017. We assessed long-term exposure to ambient PM_2.5_ using annual mean concentrations estimated from satellite-based model. We assigned time-varying annual exposures to individuals based on their residential postal code. For both types of analyses, we estimated the association with all-cause and IHD mortality using Cox proportional hazard models adjusted for age, time-varying neighborhood socioeconomic status, and current year.

**Results:**

The cohort included 7,506,027 individuals, with 996,665 and 231,376 deaths from all causes and IHD, respectively. In the between-subjects analysis, hazard ratios from linear models were 1.03 (95% confidence interval [CI]: 1.02, 1.03) for all-cause and 1.04 (95% CI: 1.03, 1.05) for IHD per interquartile range (3.3 µg/m^3^). In the within-subjects analysis, hazard ratios were 1.06 (95% CI: 1.04, 1.08) for all-cause and 1.06 (95% CI: 1.00, 1.11) for IHD per interquartile range increase in the difference between event and referent years (1.9 µg/m^3^). However, we found evidence of nonlinearity, with a steeper slope at lower concentrations.

**Conclusions:**

Consistently across the two designs, we found positive associations between annual mean exposure to low level of ambient PM_2.5_ and mortality. However, the magnitude of the association varied depending on the statistical design.

**What this study adds:**

This study is the first to use both a traditional between-subject survival analysis and a self-controlled analysis to examine the association between long-term exposure to ambient PM_2.5_ and the risk of mortality within the same population. In the context of low concentration of PM_2.5_ in Canada, we found positive associations with all-cause and ischemic heart disease mortality. However, the shape of the concentration-response functions varied depending on the design used. The fact that both designs support a positive association strengthens the case for causality, whereas the range of estimated associations contributes insights about the potential magnitude of the true effect.

## Introduction

Associations between long-term exposure to ambient fine particles (PM_2.5_) and mortality have been traditionally examined in longitudinal cohort studies where each individual with the event is compared to other individuals at risk at the time this event occurred. Such cohort studies have been conducted across numerous countries and cities, and have generally reported positive associations between long-term exposure to PM_2.5_ and mortality.^1–4^ A comprehensive systematic review and meta-analysis of 25 cohort studies has concluded that long-term PM_2.5_ exposure was significantly associated with increased mortality.^1^ However, substantial statistical heterogeneity (*I*^2^ = 89%) was found across effect estimates reported in these studies.

A number of factors have been suggested to explain this heterogeneity.^1–3^ The composition of PM_2.5_ is one factor. PM_2.5_ originates from a variety of anthropogenic and natural sources (e.g., industries, road traffic, forest fires, and residential wood burning) resulting in considerable variation in its composition, which may influence its toxicity across locations.^5–9^ Differences in study population characteristics, notably in terms of age and comorbidity, are another factor that may influence the magnitude of associations found across studies.^3,10,11^ The exposure assessment is another source of heterogeneity. Indeed, cohort studies have relied on different methods to predict pollutant concentrations, such as nearest monitoring station, land use regressions based on measurements, and satellite-based models. Furthermore, exposure models have varying performance, and concentrations are estimated at varying spatial resolution, and assigned to individuals using different spatial scales (e.g., residential address, residential postal code, and US county). Studies using high-resolution exposure assessment methods less prone to exposure errors have been shown to report higher effect estimates.^2^ Earlier work from Canada examining associations between long-term PM_2.5_ and mortality suggests that the importance of spatial scale of exposure assessment may be variable across causes of death.^12^ The consideration of different temporal window or moving averages of exposure (e.g., current annual mean exposure vs. 8 years average) may also influence effect estimates.^12^

Confounding is another factor that may contribute to the observed heterogeneity. Associations with mortality may be confounded by individual risk factors (e.g., smoking, diet, and physical activity) and local socioeconomic factors affecting health outcomes (e.g., neighborhood poverty) spatially associated with long-term ambient PM_2.5_ concentrations.^13^ Studies investigating the effect of long-term PM_2.5_ on mortality using large administrative cohorts often lack individual-level information about behavioral and lifestyle risk factors. Those studies have typically been limited to statistical adjustment for neighborhood socioeconomic status (SES) variables.^1^ However, residual or unmeasured confounding may still bias associations as individuals living in the same neighborhood (as defined by the contextual factors) may have different individual risk factor profiles.

Self-controlled approaches, which differ in their confounding control by using intraindividual contrasts, can be used to control by design for individual time-invariant or slowly varying confounders, observed or not. In such approaches, an individual’s exposure level at the time of death is compared with exposure experienced by the same individual at other times. By contrasting an individual to himself, time-invariant confounders are accounted for by design, while time-variant confounders can be addressed statistically. The time-series study design is an example of self-controlled approach that relies on aggregated data but has been extensively used to demonstrate how daily counts of death within a city correlate with daily fluctuations in air pollution levels.^14^ Analogously, the case-crossover design enables to assess the short-term effects of air pollution by contrasting the exposure just before the outcome onset with exposure during control periods supplied by each case themselves.^15,16^ When performed at the individual level, the case-crossover can allow to account for individual factors that may vary on short time scales, and which may confound or modify associations.^16^

While self-controlled designs have been extensively used for the investigation of short-term effects of air pollution, their application to long-term effects remains scarce. In this study, we aim to assess associations between long-term exposure to ambient PM_2.5_ and mortality using two distinct statistical designs: (i) a traditional survival analysis that contrasts different individuals at a similar time and (ii) a self-controlled approach that contrasts each case to themselves at different times, therefore controlling by design for time-invariant individual confounders measured or not. We carried this comparison by examining the associations between all-cause and ischemic heart disease (IHD) mortality and exposure to low concentration of PM_2.5_ in a population-based cohort in Canada.

## Methods

### Study design and population

We used an open retrospective cohort created from the Quebec Integrated Chronic Disease Surveillance System (QICDSS).^17^ The cohort includes all individuals aged 20 years or older in the province of Quebec, Canada, between 2002 and 2017. We restricted the entry in the cohort to individuals who had lived for at least 4 years in the province of Quebec. Those entering the cohort were followed until death, migration out of the province, or termination of follow-up. We focused herein on all-cause (International Classification of Diseases 10th revision [ICD-10] codes A00-Z99) and IHD (ICD-10 codes I20-I25) causes of death. All-cause mortality is equivalent to natural-cause mortality given accidental deaths account for a small proportion (<5%) of all deaths, and there is no clear evidence that air pollution is associated with accidental mortality.^1^ We studied IHD mortality because it is the top cause of mortality globally and a specific cause for which exhibit some of the strongest associations with PM_2.5_.^1,18,19^ The cohort included time-varying residential six-character postal codes, which is the most precise information available because of confidentiality reason. Person-years with invalid residential postal codes (<1%) were excluded from the analysis because air pollution exposure could not be assigned.

### Exposure assessment

We used existing annual average concentrations of ambient PM_2.5_ at each postal code for the period 2002–2017 available from the Canadian Urban Environmental Health Research Consortium (CANUE, PM_2.5_V3).^20^ Briefly, these estimates are ground-level PM_2.5_ concentrations that derive from a model combining satellite information with output from an atmospheric chemistry transport model. These estimates were then calibrated using measurements from ground-based sensors of ambient PM_2.5_ concentrations and a geographically weighted regression model. The resulting PM_2.5_ annual mean concentrations at a spatial resolution of 0.01 × 0.01 degree (~1 × 1 km) are highly correlated (*R*^2^ = 0.81) with ground measurements at fixed site monitoring stations.^20^ These gridded surface datasets were used by CANUE to assign annual mean PM_2.5_ concentrations at each Canadian postal code and each year of our study period. These yearly PM_2.5_ concentrations were linked to each of our cohort participants based on their time-varying six-character residential postal code throughout their follow-up. The exposure was, therefore, time-varying as it accounts for residential moving and yearly changes in PM_2.5_ concentration levels at each postal code.

### Covariates

Age and sex are important risk factors of mortality, which are included in the health database. As proxy of SES, we extracted neighborhood-level covariates from the Canadian censuses, including the percentage of immigrants, the median household income, and the percentage of individuals who completed high school in the dissemination area of residence. A dissemination area is a small, stable, and relatively homogeneous geographic area having a population usually varying from 400 to 700 individuals. Dissemination areas are the smallest geographical units for which socioeconomic data are available.^21^ We assigned time-varying covariates from the nearest census year (i.e., 2001, 2006, 2011, and 2016) to cohort participants based on their time-varying residential postal code.

### Statistical analysis

We assessed the association between time-varying annual mean exposure to ambient PM_2.5_ and mortality (all-cause and IHD) using two statistical designs (Figure S1; http://links.lww.com/EE/A334 shows a schematic of the two designs). First, we performed a typical survival analysis that contrasts each individual who died with other cohort subjects who were at risk of dying at the time the case died. Specifically, we used the Cox proportional hazard model with age in days as the time scale.^22^ The model was adjusted for sex and for quintiles for the dissemination area percentage of immigrants, median household income, and percentage of high school graduates. We further adjusted for the calendar year. We assessed the proportional hazard assumption through statistical testing and graphical diagnostics based on Schoenfeld residuals.

Second, we used a self-controlled approach similar to a case-crossover design used for acute effects of short-term exposure to air pollution but adapted for effects of long-term exposures.^23^ In this design, each case serves as its own control, thus allowing to control for personal time-invariant and slowly varying confounders measured or not (e.g., sex, ethnicity, smoking history, and history of occupational exposure). The hazard period was the year that the event occurred, whereas we considered each year of follow-up before the hazard period as distinct reference periods for control times. We estimated the association using a Cox proportional hazards model. Rather than using strata to define each risk set, we used time intervals that were specific to each individual and not overlapping across individuals; this approach, which is equivalent to a conditional logistic regression, leads to substantial gain in computation times for large cohort data analysis.^16^ We statistically adjusted for age and calendar year by including a linear and a quadratic term for each variable consistently with Schwartz et al.^23^ We further controlled for neighborhood SES as in the between-subjects analysis using quintiles for the dissemination area percentage of immigrants, median household income, and percentage of high school graduates.

In both analyses, we assessed potential nonlinearity of the relationship between PM_2.5_ and mortality by using natural cubic splines with three degrees of freedom. We determined the shape of the relationship based on a comparison of the Akaike information criterion of the linear and nonlinear models, and by visual inspection of the response functions. For the purpose of comparison of our findings with Schwartz et al,^23^ we also performed the self-controlled analysis for all-cause mortality restricted to individuals ≥65 years of age.

### Presentation of results

We present findings from both linear and nonlinear models. For the linear model, we report adjusted mean hazard ratios (HRs) and 95% confidence intervals (CIs) from the estimated regression coefficients for an interquartile (IQR) increment based on the distribution of annual mean concentrations experienced by the full cohort. For the self-controlled analysis, we additionally report results for an IQR increment based on the difference between the concentrations experienced during the hazard period (i.e., year of the event) and control years. This metric was argued as the relevant exposure term in the case-crossover design, thus plausibly in the self-controlled approach used herein.^24^

For nonlinear models, we present figures illustrating concentration-response curves and estimated HRs (and 95% CIs) comparing the 25th–5th percentile of the exposure distribution, as well as the 75th–25th percentile and the 95th–75th percentile.

## Results

### Description of the cohort

The cohort included a total of 7,506,027 individuals, with 996,665 and 231,376 deaths from all-cause and IHD causes, respectively (Table 1). On average, participants were followed for 13 years, for a total of 98,588,785 person-years of follow-up. The mean age at entry in the cohort was 41 years (standard deviation [sd] = 18), whereas the mean age at time of death was 68 years (sd = 15). Compared with the full cohort, cases for all-cause and IHD mortality tended to live in dissemination areas with lower median household income, higher percentage of high school graduates, and having similar neighborhood percentage of immigrants.

**Table 1.** Descriptive statistics of the cohort of adults aged ≥20 years old in the province of Quebec, Canada, 2002–2017^a^

	Full cohort (N = 7,506,027) Person-years (%)	Cases only	
		All-cause mortality (N = 996,665) Person-years (%)	Ischemic heart disease mortality (N = 231,376) Person-years (%)
Sex			
Female	51,449,312 (52.2)	4,650,802 (51.4)	929,917 (46.7)
Male	47,139,473 (47.8)	4,407,354 (48.6)	1,060,713 (53.3)
Age (years)			
20–40	31,650,410 (32.1)	219,146 (2.4)	13,728 (0.7)
40–60	37,841,671 (38.4)	1,485,107 (16.4)	234,066 (11.8)
60–80	23,605,604 (23.9)	4,388,595 (48.4)	961,764 (48.3)
≥80	5,491,099 (5.6)	2,965,308 (32.7)	781072 (39.2)
Percent of immigrants			
Quintile 1	22,928,597 (26.2)	1,971,454 (27.6)	425,592 (27.5)
Quintile 2	12,137,882 (13.9)	1,122,282 (15.8)	248,287 (16.0)
Quintile 3	17,510,512 (20.0)	1,382,706 (19.3)	302,513 (19.6)
Quintile 4	17,525,416 (20.0)	1,376,924 (19.3)	301,636 (19.5)
Quintile 5	17,517,751 (20.0)	1,286,622 (18.0)	269,129 (17.4)
Missing	10,968,627	1,918,167	443,473
Median household income			
Quintile 1	17,499,038 (20.0)	2,458,359 (34.8)	569,637 (36.8)
Quintile 2	17,499,398 (20.0)	1,750,559 (24.4)	384,296 (24.8)
Quintile 3	17,496,504 (20.0)	1,311,698 (18.3)	277,995 (18.0)
Quintile 4	17,499,424 (20.0)	992,505 (13.8)	198,671 (12.8)
Quintile 5	17,496,682 (20.0)	624,284 (8.7)	116,375 (7.5)
Missing	11,097,739	1,920,752	443,656
Percent of high school graduates			
Quintile 1	17,536,712 (20.0)	1,223,451 (17.1)	252,943 (16.3)
Quintile 2	17,599,968 (20.0)	1,172,499 (16.4)	248,907 (16.0)
Quintile 3	17,512,810 (20.0)	1,248,267 (17.5)	267,937 (17.3)
Quintile 4	17,599,717(20.1)	1,459,045 (20.4)	317,461 (20.5)
Quintile 5	17,430,888 (19.9)	2,054,233(28.7)	464,210 (29.9)
Missing	10,908,690	1,900,662	439,172

a Missing corresponds to invalid postal codes, which precluded us to assign exposure and area-based covariates. Percentages were computed for person-years contributing to the analysis (thus excluding missing). Quintile 1 corresponds to the lowest category and quintile 5 the highest.

Descriptive statistics about ambient annual PM_2.5_ exposure for the entire cohort and specifically for participants contributing to the within-subjects analysis are provided in Table S1; http://links.lww.com/EE/A334 and Figure S2; http://links.lww.com/EE/A334. Over all person-years of follow-up, the annual mean concentration of PM_2.5_ at residence of participants was on average 8.08 (sd = 2.39) µg/m^3^ for the full cohort, 8.52 (sd = 2.42) µg/m^3^ for all-cause mortality cases, and 8.55 (sd = 2.44) µg/m^3^ for IHD mortality cases (Table S1; http://links.lww.com/EE/A334). The IQR increment in exposure was 3.3 µg/m^3^ for both the full cohort and cases. Exposure in the hazard period (i.e., year of death) was on average 0.77 (sd = 1.76) µg/m^3^ and 0.73 (sd = 1.73) µg/m^3^ less than across referent periods for all-cause mortality and IHD mortality cases, respectively (Table S1; http://links.lww.com/EE/A334). The IQR of the absolute difference in ambient PM_2.5_ exposure between the hazard year and referent years was 1.90 µg/m^3^.

### Associations between annual mean PM_2.5_ and mortality

Table 2 shows estimated associations from linear models between annual mean concentrations of ambient PM_2.5_ and mortality. In the between-subjects analyses, the adjusted HRs per IQR range increase (3.3 µg/m^3^) in annual PM_2.5_ were 1.03 (95% CI: 1.02, 1.03) and 1.04 (95% CI: 1.03, 1.05) for all-cause and IHD mortality, respectively. In the self-controlled analyses, the adjusted HRs for a similar increment (3.3 µg/m^3^) were 1.10 (95% CI: 1.06, 1.15) for all-cause mortality and 1.10 (1.01, 1.20) for IHD death. For an increment of 1.9 µg/m^3^, which corresponds to an IQR in the difference between the event year and the referent period among cases only, the mean HR was 1.06 for both all-cause and IHD mortality. In the self-controlled approach, when restricting the analyses to individuals aged 65 years and older, the estimated HRs for all-cause mortality were 1.11 (95% CI: 1.06, 1.15) per IQR increase based on absolute concentration values (3.3 µg/m^3^), and 1.08 (95% CI: 1.05, 1.11) per IQR based on the difference between event and control years (1.9 µg/m^3^).

**Table 2.** Adjusted associations per interquartile range increment between annual mean exposure to ambient PM_2.5_ and mortality in adults aged ≥20 years in Quebec, Canada (2002–2017)

Models	IQR (µg/m^3^)	Association per IQR (95% confidence interval)
Between-subject analysis^a^		
All-cause mortality	3.3	1.03 (1.02, 1.03)
Ischemic heart disease mortality	3.3	1.04 (1.03, 1.05)
Self-controlled analysis^b^		
All-cause mortality	3.3	1.10 (1.06, 1.15)
	1.9^c^	1.06 (1.04, 1.08)
Ischemic heart disease mortality	3.3	1.10 (1.01, 1.20)
	1.9^c^	1.06 (1.00, 1.11)

a Models adjusted for age, sex, calendar year, and neighborhood SES covariates (i.e., quintiles of % of immigrants, % of high school graduates, median household income).

b Models adjusted for age (linear and quadratic terms), calendar year (linear and quadratic terms), and neighborhood SES covariates (i.e., quintiles of % of immigrants, % of high school graduates, median household income). Time-invariant factors are controlled by design.

c Corresponds to the IQR of the within-subject difference between event and referent years.

**Figure 1.**
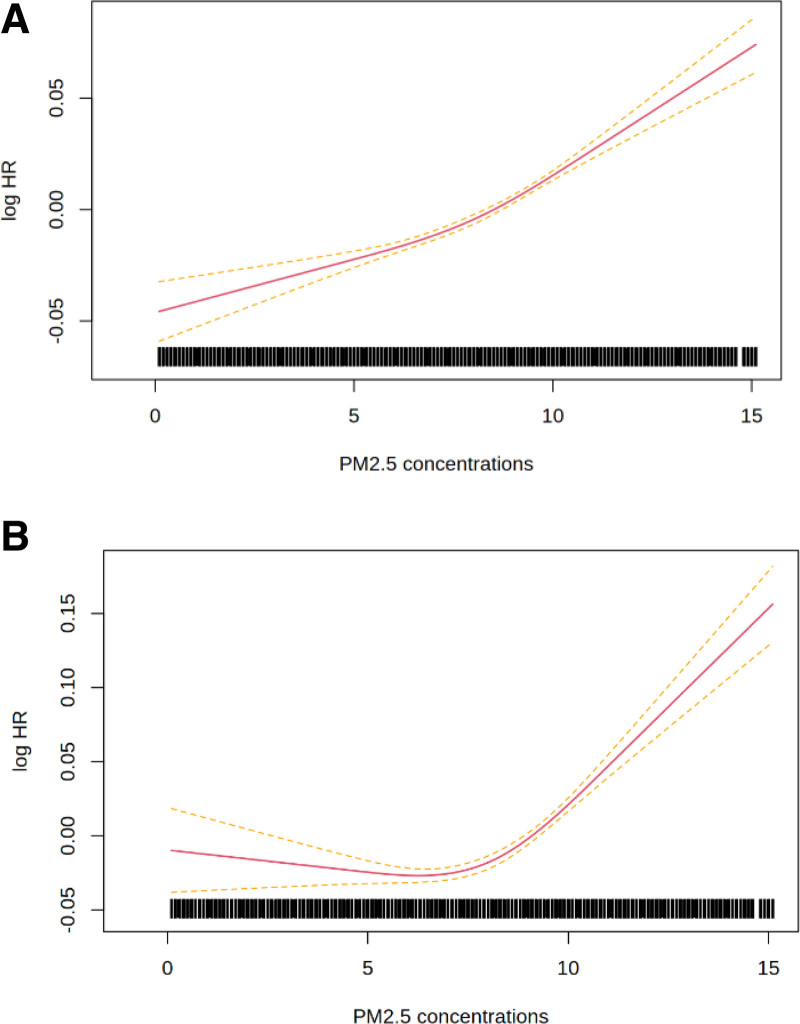
Between-subject analysis response functions between annual mean exposure to ambient PM_2.5_ (µg/m^3^) and the risk of (A) all-cause mortality and (B) ischemic heart disease mortality in adults aged ≥20 years in Quebec (Canada). The response functions were fitted using natural cubic splines with three degrees of freedom (knots located at 10th, 50th, and 90th percentiles of exposure). Red lines represent the mean log hazard ratios of the nonlinear functions, and the dashed yellow lines represent the 95% confidence intervals. Models are adjusted for age, sex, calendar year, and quintiles of median household income, percentage of immigrants and of high school graduates.

**Figure 2.**
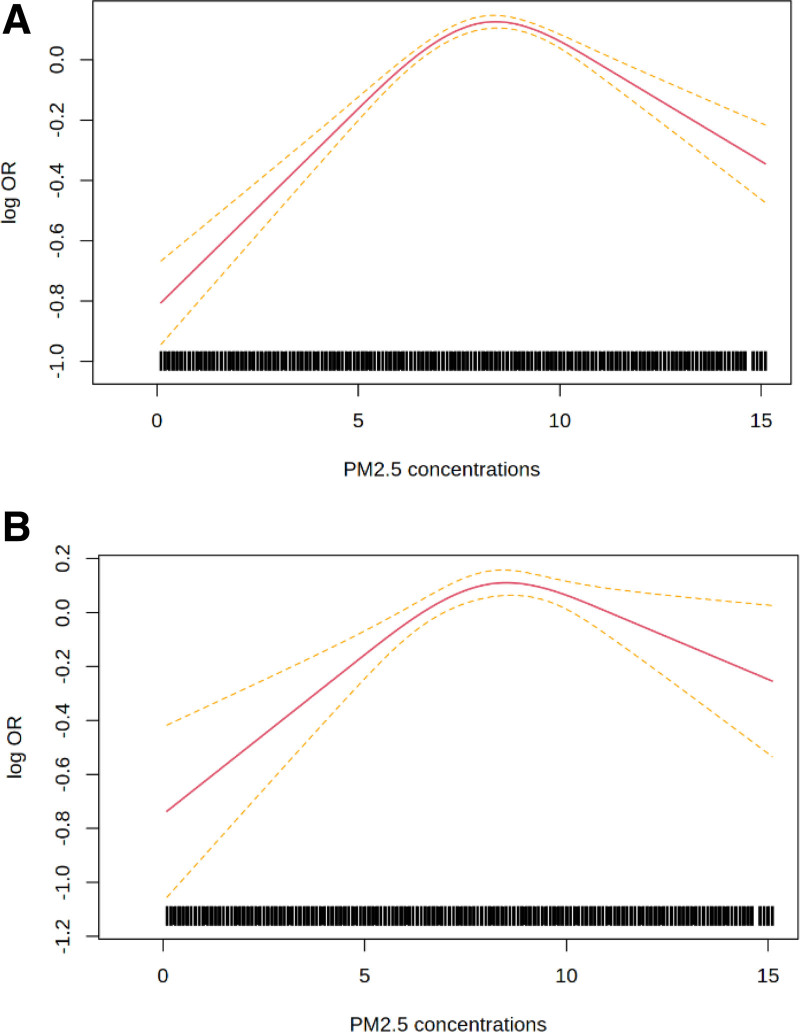
Within-subject analysis response functions between annual mean exposure to ambient PM_2.5_ (µg/m^3^) and the risk of (A) all-cause mortality and (B) ischemic heart disease mortality in adults aged ≥20 years in Quebec (Canada). The response functions were fitted using natural cubic splines with three degrees of freedom (knots located at 10th, 50th, and 90th percentiles of exposure). Red lines represent the mean log odds ratio (OR) of the nonlinear functions, and the dashed yellow lines represent the 95% confidence intervals. Models are adjusted for age (linear and quadratic terms), calendar year (linear and quadratic terms), and quintiles of median household income, percentage of immigrants and of high school graduates. Time-invariant factors are controlled by design.

Figures 1 and 2 show the concentration-response functions fitted using splines, with Akaike information criterion statistics reported in Table S2; http://links.lww.com/EE/A334. Numerical values of the adjusted associations for selected changes in exposures are reported in Table 3. In the between-subjects analyses (Figure 1), we found evidence of departure from linearity for IHD but not for all-cause mortality. For IHD, the response function depicted no association until approximately 7 µg/m^3^. In the self-controlled approach (Figure 2), models using splines displayed improved fit over the linear models. Both curves showed a steep slope until approximately the 75th percentile of the exposure distribution from which the slopes flatten and become negative at high ranges (Figure 2). A change from the 25th to the 5th percentile of the exposure distribution yielded HRs of 1.40 (95% CI: 1.33, 1.48) for all-cause mortality and 1.36 (95% CI: 1.20, 1.54) for IHD death (Table 3).

**Table 3.** Adjusted associations between annual mean exposure to ambient PM_2.5_ and mortality in adults aged ≥20 years in Quebec, Canada (2002–2017), fitted as natural cubic splines with 3 degrees of freedom

Model/cause of death	Adjusted associations (95% CI) per percentile changes in exposure		
	25th vs. 5th percentile	75th vs. 25th percentile	95th vs. 75th percentile
	(6.5 vs. 3.8 µg/m^3^)	(9.8 vs. 6.5 µg/m^3^)	(11.8 vs. 9.8 µg/m^3^)
Between-subject analyses^a^			
All-cause	1.01 (1.01, 1.02)	1.03 (1.02, 1.03)	1.02 (1.02, 1.03)
Ischemic heart disease	0.99 (0.98, 1.01)	1.04 (1.04, 1.05)	1.05 (1.04, 1.06)
Within-subject analyses^b^			
All-cause	1.40 (1.33, 1.48)	1.06 (1.02, 1.10)	0.86 (0.82, 0.89)
Ischemic heart disease	1.36 (1.20, 1.54)	1.07 (0.98, 1.17)	0.89 (0.81, 0.97)

a Models adjusted for age, sex, calendar year, neighborhood SES covariates (quintiles of % of immigrants, % of high school graduates, and median household income).

b Models adjusted for age (linear and quadratic terms), calendar year (linear and quadratic terms), and neighborhood SES covariates (quintiles of % of immigrants, % of high school graduates, median household income). Time-invariant factors are controlled by design.

## Discussion

This study is the first to examine the association between long-term exposure to ambient PM_2.5_ and the risk of mortality using both a traditional survival analysis (between-subjects analysis) and a self-controlled analysis (within-subjects analysis) within the same population. Consistently across designs, we found positive associations between annual PM_2.5_ exposure and the risk of mortality, both all-cause and IHD, which is the top cause of mortality globally.^19^ The magnitude of the linear associations was slightly greater in the self-controlled approach as compared with the estimates from the traditional between-subjects analyses. However, in nonlinear models, we found that the shape of the concentration-response function differs between the two analytical designs. Notably, the slopes were steeper at lower PM_2.5_ concentrations in the within-subjects analyses as compared with the between-subjects traditional analyses.

Differences in confounding control methods between the two analytical designs may have contributed to observed variations in concentration-response functions. While we cannot ascertain which method provides the most valid estimate, the self-controlled approach benefits from controlling by design for time-invariant confounders, observed or not. This includes host characteristics, such as genetic or preexisting comorbidity, and any behavioral risk factors or environmental exposures that occurred before the start of follow-up, including smoking and occupational exposure history, obesity, and lifetime dietary habits. To confound the within-subjects association, a risk factor would have to vary across years in a way that was not captured by the linear and quadratic terms we used to model time trend and age, as well as by other adjustments considered in our regression models. While it remains uncertain whether effects accumulate over time or whether there are sensitive periods during the life course where exposures may lead to stronger impacts on health, exposure occurring before the follow-up is controlled by design. In contrast, these factors are hardly addressed in traditional cohort studies with between-subjects contrast.

Other differences between the two analytical designs deserve to be highlighted. While the two analytical designs aim to estimate the association between long-term PM_2.5_ exposure and the risk of death, conceptually, they address this question by using different counterfactuals.^25^ In the traditional survival analysis, the contrast is between people and the underlying question is “Why this person died and not the others?.” The within-subjects analysis compares the same person across years, thus addressing the question “Why this person died now?.” As a result, the study base differs between the two approaches with the self-controlled analysis being a case-only design, thus involving only individuals who died. In terms of exposure, the traditional survival analysis relies mainly on spatial contrasts across study participants. In the self-controlled approach, the exposure contrast between the hazard and the referent periods is dominated by temporal variations (participants contributing to the within-subjects analysis moved on average 0.9 times during their follow-up; Table S3; http://links.lww.com/EE/A334). Because the temporal variations in PM_2.5_ are smaller than its spatial variations, this yielded decreased statistical power in the self-controlled design, resulting in larger CIs as compared with the between-subject analysis.

In a comprehensive systematic review and meta-analysis of cohort studies examining the association between long-term exposure to PM and mortality, pooled risk ratios per 10 µg/m^3^ increase in long-term ambient PM_2.5_ was 1.08 (95% CI: 1.06, 1.09; N = 25 studies) for all-cause mortality and 1.16 (95% CI: 1.10, 1.21; N = 22 studies) for IHD mortality; however, for both outcomes, there was high heterogeneity across study findings (*I*^2^ = 89% and 78%, respectively). Consistently, our between-subject analyses using annual exposures yielded for a similar increment HRs of 1.09 (95% CI: 1.07, 1.10) and 1.12 (95% CI: 1.10, 1.15) for all-cause and IDH death, respectively. Our results are also in agreement with most recent large cohort studies from North America and Europe that reported increased risk of all-cause (or equivalently nonaccidental) and cardiovascular-related mortality with long-term PM_2.5_ exposure, even at low concentrations.^18,26,27^ Notably, in the largest Canadian cohort study (the Stacked CanCHEC) representing nearly 1.3 million deaths, Brauer et al^18^ used a 10-year moving average exposure and 1-year lag and they found HRs in linear models of 1.08 for nonaccidental mortality (95% CI: 1.07, 1.10) and 1.23 (95% CI: 1.20, 1.26) for IDH mortality (215,700 deaths) per 10-μg/m^3^ increase. Compared with our result, the elevated association found by Brauer et al^12^ for IHD could be due to the use of a longer moving average period, which was shown to result in stronger associations between PM_2.5_ and IHD mortality. PM_2.5_ sources and composition which likely influence toxicity is another possible reason for the difference in IHD associations.

To our knowledge, only one study has applied a self-controlled design to investigate the long-term effects of ambient PM_2.5_.^23^ This study, conducted in the United States among individuals aged 65 years and older from the Medicare cohort, found a 5.4% (95% CI: 4.7, 6.1) increase in mortality per 5 µg/m^3^ increase in exposure. However, in individuals whose exposure was below 12 µg/m^3^ (i.e., the current U.S. air quality standard), the estimated risk was 12.7% (95% CI: 11.3, 14.2). For a similar exposure increment, we found, in the context of exposure to low concentration of PM_2.5_ (95th percentile of the exposure distribution = 12.2 µg/m^3^) in Canada, a 23.0% (95% CI: 14.9, 31.6) increase in all-cause mortality in the elderly (≥65 years old). It should be noted that the authors reported associations for an exposure increment based on the distribution of exposures across all individuals. Herein, we further report associations based on distributional properties of the concentration difference between event and control years, which we consider to be the relevant metric of exposure as in the case-crossover.^24^ Reporting findings for an IQR range based on the distribution of absolute concentration values across subjects may be misleading and will likely overestimate the effects of air pollution as between-subjects is greater than within-subjects variation.

Large cohorts based on medico-administrative data are increasingly used. These cohorts minimize selection bias and confer high statistical power, albeit at the expense of limited information on possible confounding factors. Even when available, information collected through survey may be subject to information bias. Some studies examining the association between long-term ambient PM_2.5_ and mortality have used generalized propensity scores and doubly robust methods as alternative approaches to address confounding.^28–30^ These alternative approaches yielded similar results to traditional Cox models, suggesting that confounding control methods may not be a meaningful source of heterogeneity. However, unlike the self-controlled design used, which addresses confounding mostly by design, these alternative approaches rely on between-subjects contrast and statistical adjustments, thus making them possibly more prone to residual and unmeasured confounding.

An important strength of our study lies in our cohort constructed from the health administrative data that minimizes selection bias by capturing 99% of the Quebec population.^17^ We assigned exposure to ambient PM_2.5_ using the residential postal codes, which is the most precise level of information available. However, exposure estimates were residential and not personal, as we could not account for time-activity patterns. Overall, misclassification of the exposure is expected to be nondifferential, thus biasing our estimates toward the null. The traditional between-subject survival analyses were limited in terms of individual information about risk factors that could be spatially associated with ambient PM_2.5_ concentrations, such as smoking. However, many of these personal risk factors are unlikely to be correlated with outdoor concentration of PM_2.5_, which act as an instrumental variable for personal exposure.^31^ Previous Canadian cohort studies using indirect adjustments showed that unmeasured confounding by smoking and obesity had very little effect on the estimated association between air pollution and mortality.^32–34^ In the self-controlled approach, while time-invariant or slowly varying confounders are controlled by design, we still cannot rule out residual confounding by some risk factors that may vary on shorter time scales.

## Conclusions

In this population-based cohort study, annual mean exposure to low ambient PM_2.5_ concentrations was associated with the risk of all-cause and IHD mortality in both a traditional survival analysis (between-subjects analysis) and a self-controlled analysis (within-subjects analysis). However, the magnitude and shape of the concentration-response functions varied depending on the statistical design used. Although we cannot ascertain which method provides the best estimate, the fact that both support a positive association strengthens the case for causality and contributes insights regarding the potential range of magnitude of the effect.

## Conflicts of interest statement

The authors declare that they have no conflicts of interest with regard to the content of this report.

## Acknowledgments

Mean annual ambient PM_2.5_ concentrations, indexed to DMTI Spatial Inc. postal codes, were provided by CANUE (Canadian Urban Environmental Health Research Consortium).

## Supplementary Material
