# Supplementary material for: Long-term exposure to ambient PM2.5 and mortality: A comparison of between-subjects and within-subjects survival analysis
Source: Environ Epidemiol. 2025 Mar 14;9(2):e378. doi: 10.1097/EE9.0000000000000378 (PMC11913414; doi:10.1097/EE9.0000000000000378)
Supplement: Supplementary file 1 [file ee9-9-e378-s001.pdf]

Supplementary Material

Supplemental Material

Long-term exposure to ambient PM2.5 and mortality: A comparison of between- and within-subjects survival analysis

Authors: Martin Resua Rojas[1,2,3], Julien Vachon[2,3], Elhadji Anassour-Laouan Sidi[1], Claudia Blais[1,4], Ying Liu[2,3], Audrey Smargiassi[1,2,3], Stephane Buteau[1,2,3]

Author's affiliations:

[1] Institut national de santé publique du Québec, Montreal, Canada.

[2] Department of Environmental and Occupational Health, School of Public Health, University of Montreal, Montreal, Canada.

[3] Public Health Research Center (CReSP), University of Montreal, Montreal, Canada.

[4] Faculty of Pharmacy, Laval University, Quebec, Canada.

Correspondence:

Stephane Buteau

Mailing address: C.P. 6128 Succ. Centre-ville, Montréal (Qc), H3C 3J7, Canada

Email: stephane.buteau@umontreal.ca

FIGURES

(A)

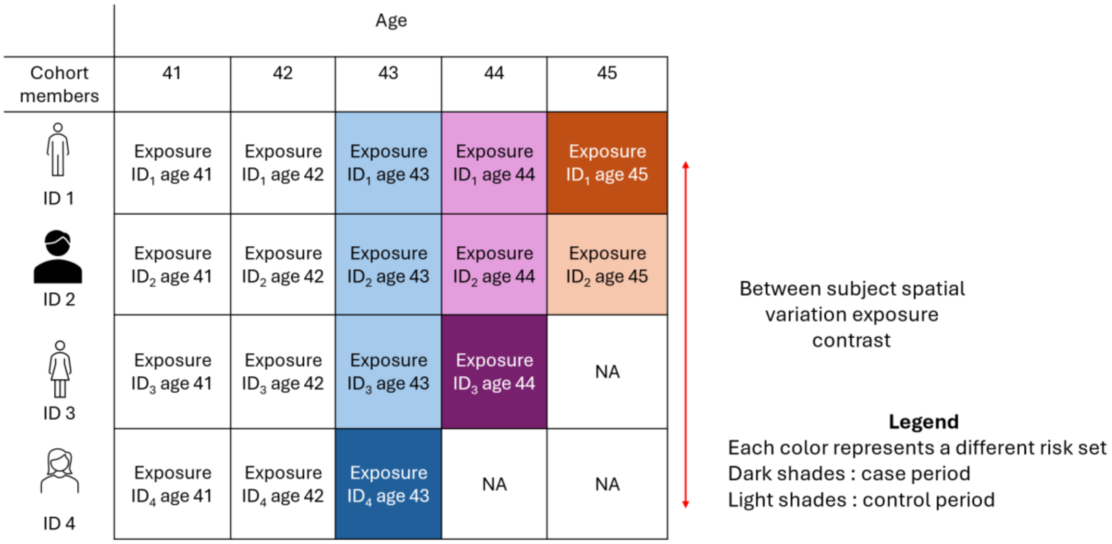

(B)

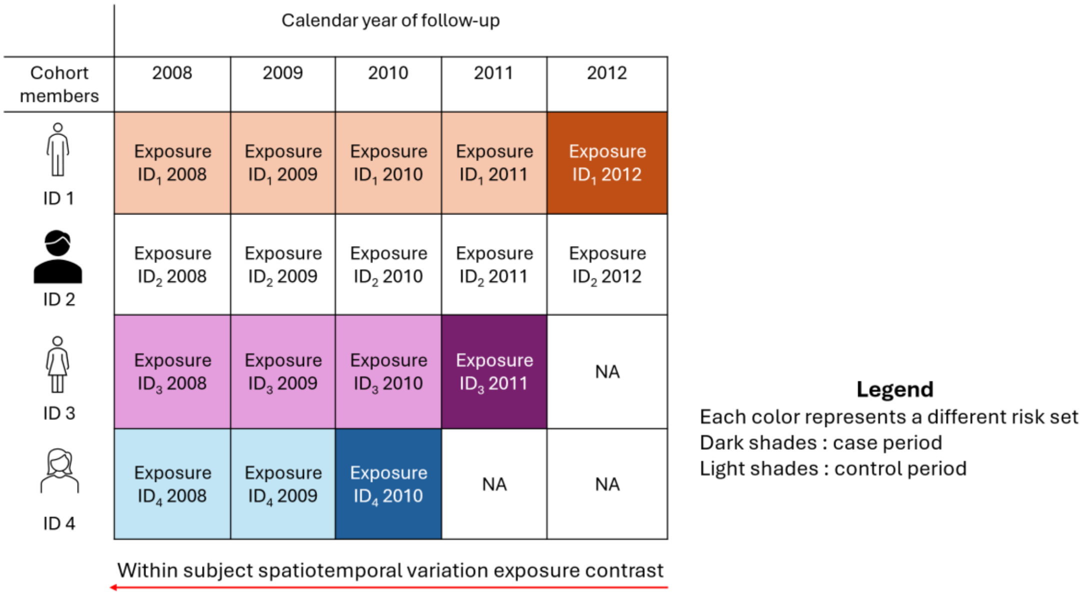

Figure S1. Schematic of (A) the between-subject and (B) the within-subjects (or self-controlled) analysis. For both designs, each color represents a different risk set, with dark shades representing the case (or hazard) period, and light shades control periods. In the between-subject analysis (A), risk sets include all individuals who were at risk at the time an event occurred. Specifically, as our Cox model used age as the timescale, risk set were built so that controls were the exact same age as the case when he

suffered the event. The analysis contrasts the annual mean $PM_{2.5}$ exposure of the case at the time of the event to the annual mean exposure of other individuals contributing to the risk set. For example, individual 4 (ID 4) who died at 43 years old would be contrasted to ID 1, 2 and 3 at time they were also 43 years old. In the within-subject analysis (B), an individual is compared to himself at different times. Specifically, for each case, the risk set includes every single year of follow-up of that individual before he suffered the event. The analysis contrasts the individual's annual mean exposure on the year he suffered the event, with annual mean exposures during each previous year of follow-up treated as distinct control periods. For example, for individual 1 (ID 1) who died in 2012, the analysis would contrast the annual mean exposure in 2012 (case period) with each of the four previous follow-up years, treated as four distinct control periods (2008, 2009, 2010 and 2011).

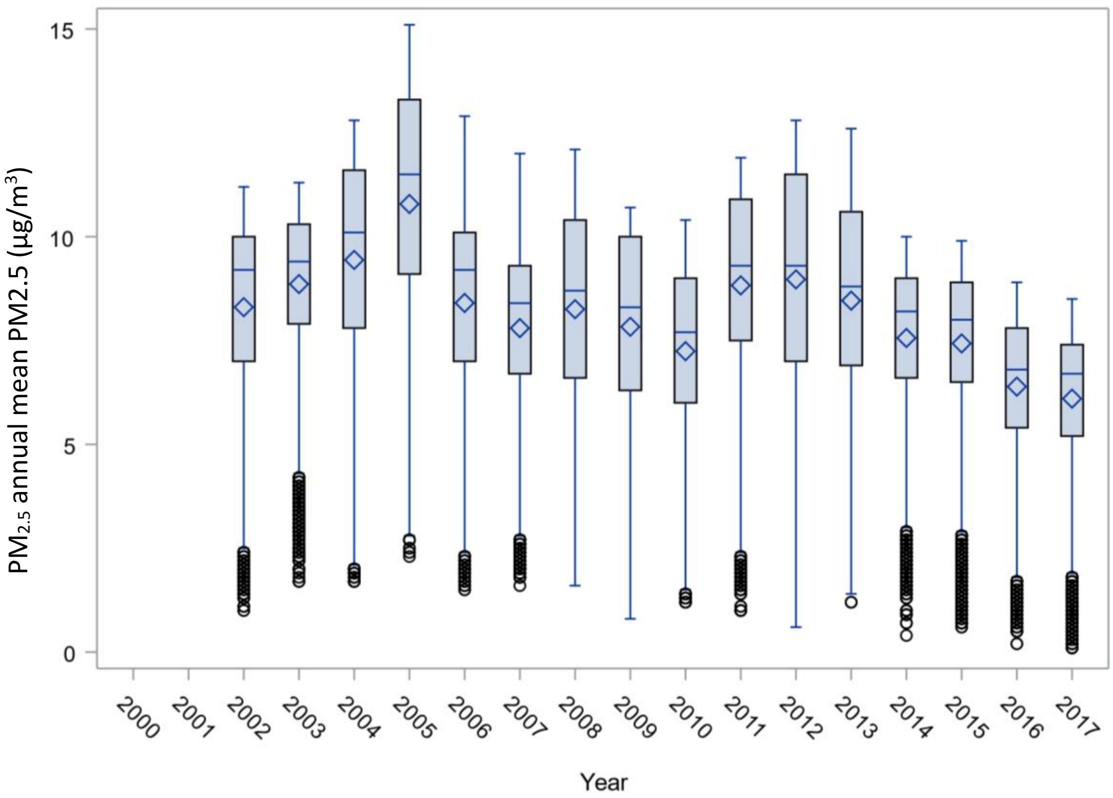

Figure S2. Boxplot showing the distribution of annual mean $PM_{2.5}$ exposure (in µg/m^3) across follow-up years in participants contributing to the within-subjects analysis. The horizontal line inside each box represents the median, whereas the diamond indicates the mean. The bottom and top of the boxes represent the first and third quartiles, respectively. The whiskers represent the first and third quartile ±1.5-fold of the interquartile range. The circles represent exposure values below the lower whisker.

TABLES

Table S1. Descriptive statistics of ambient annual $PM_{2.5}$ exposure (µg/m^3).

	Mean (sd)	Percentiles of the distribution				
		5th	25th	50th	75th	95th
All participants	8.08 (2.39)	3.8	6.5	8.2	9.8	11.8
Cases only						
All-cause mortality	8.52 (2.42)	4.0	6.9	8.9	10.2	12.2
Ischemic heart disease mortality	8.55 (2.44)	4.0	6.9	8.9	10.2	12.2
Cases only - Difference in exposure between event year and referent years[a]						
All-cause mortality	-0.77 (1.76)	-1.9	-0.1	0.6	1.8	3.9
Ischemic heart disease mortality	-0.73 (1.73)	-1.9	-0.2	0.5	1.7	3.8

Abbreviations: sd, standard deviation.

[a]The difference between exposure in the case year and exposure in previous years of follow-up was calculated for each case, then averaged across all cases.

Table S2. Model fit of the adjusted response functions for long-term exposure to ambient $PM_{2.5}$ fitted using linear and nonlinear structures in the analysis of the associations with all-cause and ischemic heart disease mortality.

**Functional form for $PM_{2.5}$**	**Akaike information criterion (AIC)**	
	**All-cause mortality**	**Ischemic heart disease mortality**
Between-subjects analyses		
Linear	13,349,015	2,959,152
Natural cubic splines, 3 dfs	13,349,004	2,959,095
Within-subjects analyses		
Linear	102,797	22,536
Natural cubic splines, 3 dfs	102,670	22,520

Abbreviations: df, degree of freedom;

Table S3. Descriptive statistics related to moving in the cohort.

	Mean (sd)	Percentiles of the distribution			
		25th	50th	75th	95th
All participants	1.8 (2.6)	0	1	3	6
Cases only – All cause	0.9 (1.7)	0	0	1	4
Cases only – Ischemic heart disease	0.9 (1.5)	0	0	1	3

Abbreviations: sd, standard deviation.
